# Curative Embolization of Small Brain Arteriovenous Malformations by Ethyl Vinyl Alcohol Copolymer: A Systematic Review and Meta-Analysis

**DOI:** 10.7759/cureus.27219

**Published:** 2022-07-25

**Authors:** Sharad Ghatge, Pratik Itti

**Affiliations:** 1 Interventional Radiology, Grant Government Medical College and Sir Jamsetjee Jeejebhoy (JJ) Group of Hospitals, Mumbai, IND; 2 Radiology, Grant Government Medical College and Sir Jamsetjee Jeejebhoy (JJ) Group of Hospitals, Mumbai, IND

**Keywords:** spetzler-martin, complete cure rate, embolization, onyx, arteriovenous malformation

## Abstract

The right choice in treating small (Spetzler-Ponce grade A) brain arteriovenous malformations (AVMs) is a matter of debate with varying views from neurology, neurosurgery, and interventional neuroradiology points of view. The Spetzler-Martin 1 and 2 brain AVMs, especially those in eloquent and deep areas that are difficult to access by micro-neurosurgery, are most suitable for a complete cure by endovascular embolization with ethyl vinyl alcohol (EVOH)-based agents.

A literature search was done with keywords such as endovascular embolization of small brain AVM. Data from 13 articles are included in the study based on predetermined inclusion and exclusion criteria.

Meta-analysis for the complete cure rate was done, publication bias was removed, and regression analysis showed a 76% cure rate with a 95% confidence interval (CI). Major complications were hemorrhage and neurological deficit, which ranged from 0-20% and 0-16% with a mean proportion of 0.11 and 0.09, respectively. Long-term (3-6 months) follow-up data showed 0-4% recurrence at three months, 0-8% recurrence at six months, and 2-10% permanent disability. The mortality rate ranged from 3% to 4%. Three illustrative cases with data from the author’s institute are included in the article. To conclude, endovascular embolization for small brain AVMs is a satisfactory treatment modality, however, prospective registries and randomized controlled trials involving embolization versus neurosurgery and/or stereotactic radiosurgery (SRS) may validate the role of embolization in small brain AVMs as curative treatment.

## Introduction and background

Introduction

The management of arteriovenous malformations (AVMs) of the brain is still not a completely settled issue in the domain of neurological care. On one hand, there is universal consensus about curative treatment of ruptured brain AVMs to avoid re-rupture and potential disability or death [1-2]. But here too, the choice of the best modality for the treatment can be reasonably argued. On the other hand, treatment of an unruptured AVM with the best medical management alone has been favored by the "A Randomized trial of Un-ruptured Brain Arteriovenous malformations (ARUBA)" trial. However, this has been marred by controversies, and critics have generally accepted the treatment of low-grade AVMs by neurosurgery, and deep, eloquent AVMs by radiosurgery. Endovascular embolization is mostly used as an adjuvant to neurosurgery and/or radiosurgery [3-4]. Small brain AVM in our study referred to Spetzler-Ponce grade A, which includes Spetzler-Martin grades 1 and 2. Here, a systematic review and meta-analysis of the literature is reported to assess the curative potential of embolization for small brain AVMs by ethyl vinyl alcohol copolymer (EVOH). A level of evidence was attached to each study. Meta-analysis for complication rate and long-term follow-up outcomes was not done because of their heterogeneity in the included studies. However, a meta-analysis of the complete cure rate was done, as the results presented in selected studies appeared suitable for applying the statistical method. Hence, the complications and long-term follow-up outcomes were statistically mentioned as mean and range.

Materials and methods

Study Design

**Figure 1:**
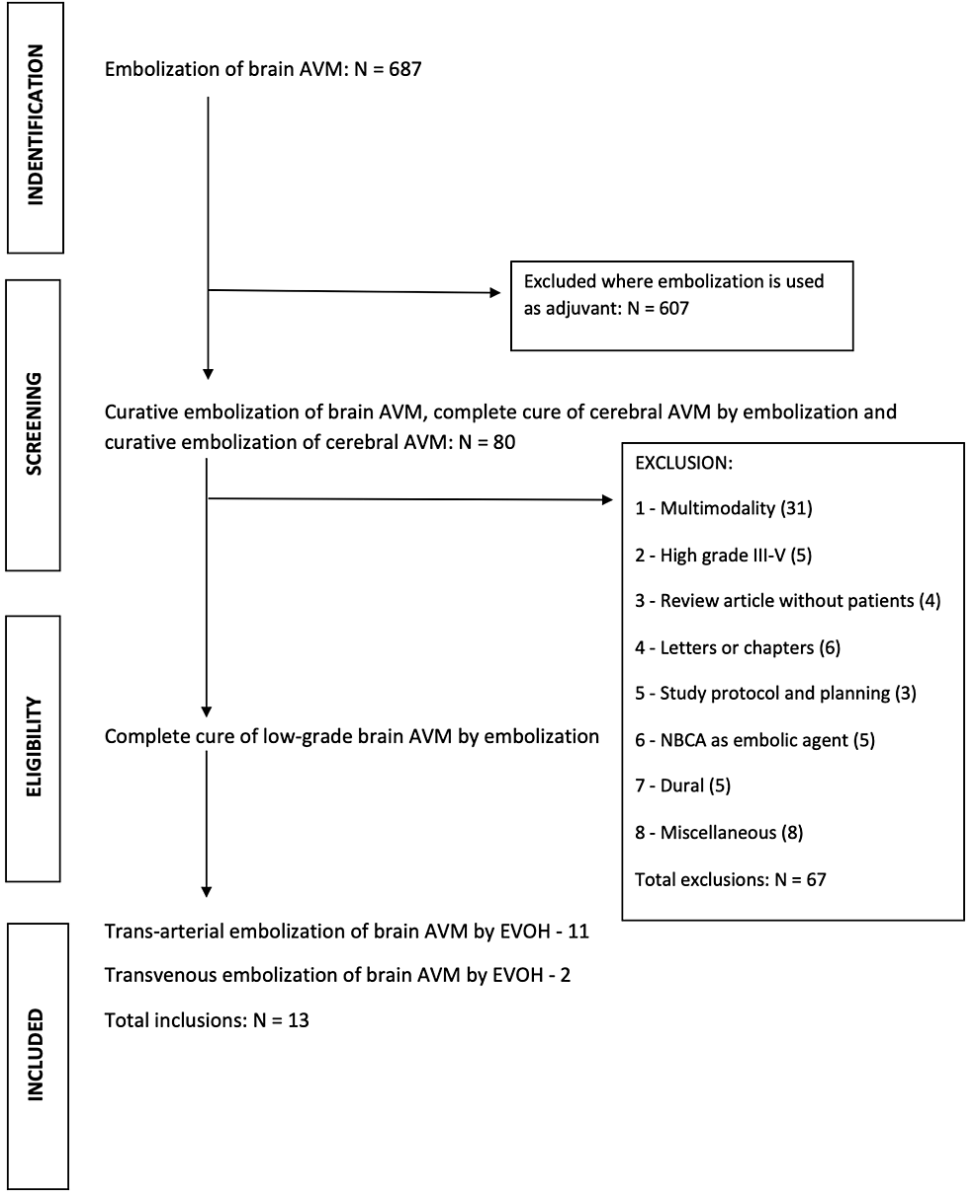
Flow chart depicting the study design AVM - arteriovenous malformation; EVOH - ethyl vinyl alcohol; NBCA - N-butyl cyanoacrylate

Our objective was to review the literature and do a meta-analysis as per the PRISMA (Preferred Reporting Items for Systematic Reviews and Meta-Analyses) guidelines in the context of curative embolization of small brain AVMs by EVOH. We searched the online databases PubMed, Embase, Science Direct, and Medline. Out of 687 articles with the term embolization of brain AVM, we have selected 80 articles with terms 1 - curative embolization of brain AVM, 2 - curative embolization of cerebral AVM, 3 - complete cure of cerebral AVM by embolization, and/or 4 - embolization as primary treatment for brain AVM. Then, the pre-determined inclusive criterion of complete cure of small brain AVM was applied by excluding articles with multiple modalities of treatment, high-grade AVM, review without patient subjects, letters, chapters, studies with planning and protocol, N-butyl cyanoacrylate (NBCA) as the embolic agent, treatment of dural arteriovenous fistula (AVF), the vein of Galen, and miscellaneous articles, totaling 67. This results in 13 articles, out of which 11 were by the transarterial route and two were by the transvenous route (Figure 1).

Acquisition of Data

From these 13 articles, we acquired the following data: number of subjects, embolization sessions, embolization procedure, complete cure rate, complication rate, and long-term follow-up outcome. The data from the author's institute is mentioned along with three illustrative cases.

Data from the author's institute: The number of patients was six from June 2021 to February 2022.

Complete obliteration rate: 93.75%

Complications: 18.75% (neurological deficit)

Deaths: 0

Analysis of the data: A systematic review and meta-analysis were done by tabulating the findings in Microsoft Excel (Microsoft Corporation, Redmond, WA). Data were analyzed using R software version 4.1.2 (R Core Team (2020). R Foundation for Statistical Computing, Vienna, Austria). Heterogeneity in the studies was assessed by using Cochran’s Q test. The derSimonian-Laird estimator was used to estimate tau squared. Egger’s test was used to check the publication bias among the studies. The data collected from included studies are tabulated in Table 1.

**Table 1:** Data extraction table

Serial number	Article	Number of patients	Number of embolization sessions	Embolization approach	% of complete cure	% of complications	Long-term follow-up outcome (%)	% of mortality
1	Lopes et al. (2016) [5]	39	39	Transarterial	80	8; neurodeficit	--	
2	Rooij et al. (2012) [6]	24	24	Transarterial	100	0	3m; 4.35 - recurrence	4.17
3	Strauss et al. (2013) [7]	92	177	Transarterial	37	8.4; hemorrhage	6m, 6.5; permanent disability	
4	Castro-Afonso et al. (2016) [8]	23	45	Transarterial	91.3	13; hemorrhage	8.7 - recurrence at 6m	
5	Abud et al. (2011) [9]	17	24	Transarterial	94.12	11.76; hemorrhage	3m, 5.88 - permanent disability	
6	Maimon et al. (2010) [10]	43	76	Transarterial	37.2	16.3; hemorrhage	-	
7	Katsaridis et al. (2008) [11]	101	219	Transarterial	53.9	8; neurodeficit	6m, 0 - recurrence	3
8	Xianli et al. (2017) [12]	6	6	Transarterial	83.3	16.67; neurodeficit	6m, 0 - recurrence	
9	Elewa et al. (2018) [13]	21	43	Transarterial	42.9	9.3; hemorrhage,	6m, 4.76 – permanent disability	
10	Mendes et al. (2018) [14]	40	41	Transvenous	92.6	5; hemorrhage	2.5; 6m: permanent disability, 0 - recurrence	
11	Mathews et al. (2007) [15]	5	19	Transarterial	100	0	-	
12	Yingkun et al. (2018) [16]	10	10	Transvenous	90	20; hemorrhage	3m, 0 - recurrence; 10 – permanent disability	
13	Andreou et al. (2008) [17]	25	26	Transarterial	84.6	12; neurodeficit	2 - recurrences at 6m	

## Review

Results

The data contain 13 studies. Table 2 gives a quantitative summary of the studies for complete cure.

**Table 2:** Quantitative summary of the studies for complete cure

Articles	Proportion (Complete cure rate)	95% CI
Mendes et al. (2018) [14]	0.925	(0.7918, 0.9756)
Mathews et al. (2007) [15]	1	(0.3782, 0.9950)
Maimon et al. (2010) [10]	0.3721	(0.2420, 0.5238)
Strauss et al. (2013) [7]	0.3696	(0.2774, 0.4723)
Xianli et al. (2017) [12]	0.8333	(0.3687, 0.9772)
Katsaridis et al. (2008) [11]	0.5347	(0.4373, 0.6294)
Rooij et al. (2012) [6]	1	(0.7487, 0.9988)
Castro-Afonso et al. (2016) [8]	0.913	(0.7112, 0.9782)
Yingkun et al. (2018) [16]	0.9	(0.5328, 0.9861)
Abud et al. (2011) [9]	0.9412	(0.6797, 0.9918)
Elewa et al. (2018) [13]	0.4286	(0.2401, 0.6403)
Lopes et al. (2016) [5]	0.7949	(0.6404, 0.8940)
Andreou et al. (2008) [17]	0.84	(0.6431, 0.9386)

From the meta-analysis, we observe that I^2^ is 84.5% (74.9.1%, 90.4%) and tau-squared (estimate of between-study variance) is observed to be 1.0258 (0.5484, 5.1550).

From Cochran’s Q test, we observe that there is significant heterogeneity in the data (p-value<0.001).

**Figure 2:**
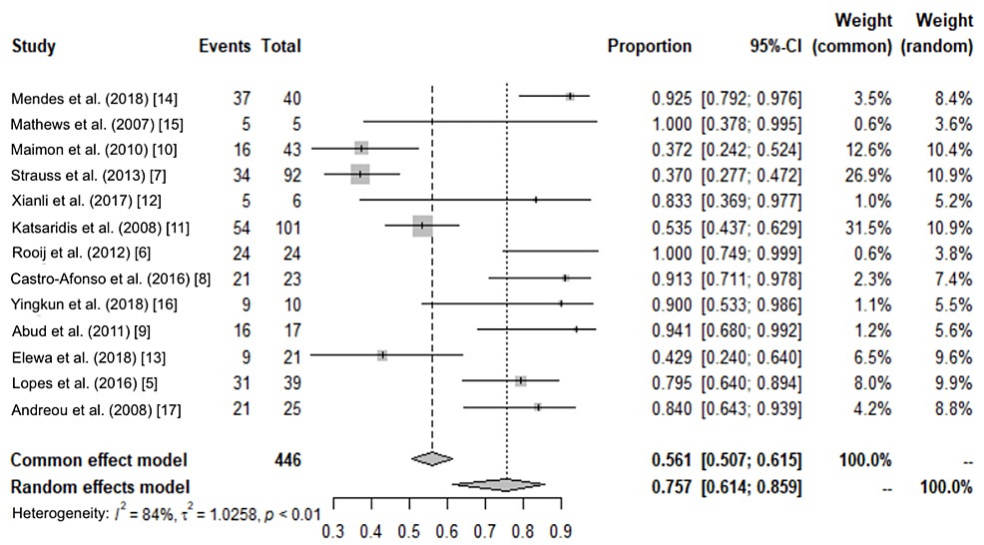
Forest plot

The following graph (Figure 2) shows the forest plot that contains information on the heterogeneity test, proportions, and confidence intervals.

Table 3 gives the results of Egger’s regression test for publication bias.

**Table 3:** Results of Egger’s regression test Abbreviation: * indicates statistical significance.

	Intercept (95% CI)	t-value	p-value
Egger’s test	3.365 (1.74 - 4.99)	4.05	0.0019*

**Figure 3:**
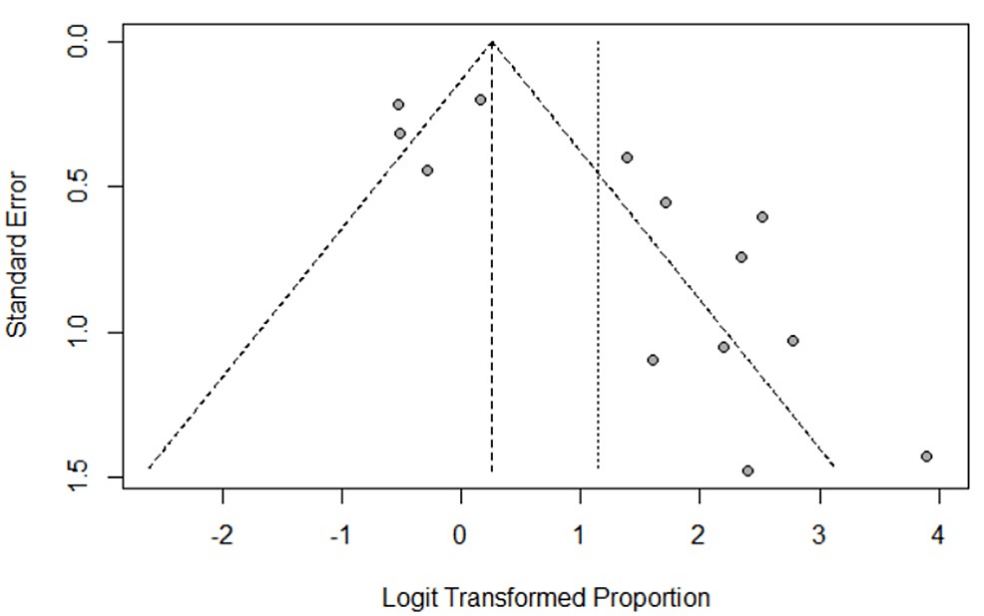
Funnel plot for publication bias

Eggers' test indicates the presence of funnel plot asymmetry. There is significant publication bias in the study and the estimated bias is 3.3654. The following graph (Figure 3) gives the funnel plot.

From the funnel plot, we observed that some points are outside the funnel, indicating that there is an asymmetric distribution of studies. Hence, we can say that a publication bias is present.

Outlier analysis was carried out. Maimon, Strauss, Katsaridis, and Elewa are found to be the outliers. The outliers were removed, and the meta-analysis was carried out on the remaining nine studies. Table 4 gives the quantitative summary of the studies.

**Table 4:** Quantitative summary of the studies

Articles	Proportion (Complete cure rate)	95% CI
Mendes et al. (2018) [14]	0.925	(0.7918, 0.9756)
Mathews et al. (2007) [15]	1	(0.3782, 0.9950)
Xianli et al. (2017) [12]	0.8333	(0.3687, 0.9772)
Rooij et al. (2012) [6]	1	(0.7487, 0.9988)
Castro-Afonso et al. (2016) [8]	0.913	(0.7112, 0.9782)
Yingkun et al. (2018) [16]	0.9	(0.5328, 0.9861)
Abud et al. (2011) [9]	0.9412	(0.6797, 0.9918)
Lopes et al. (2016) [5]	0.7949	(0.6404, 0.8940)
Andreou et al. (2008) [17]	0.84	(0.6431, 0.9386)

**Figure 4:**
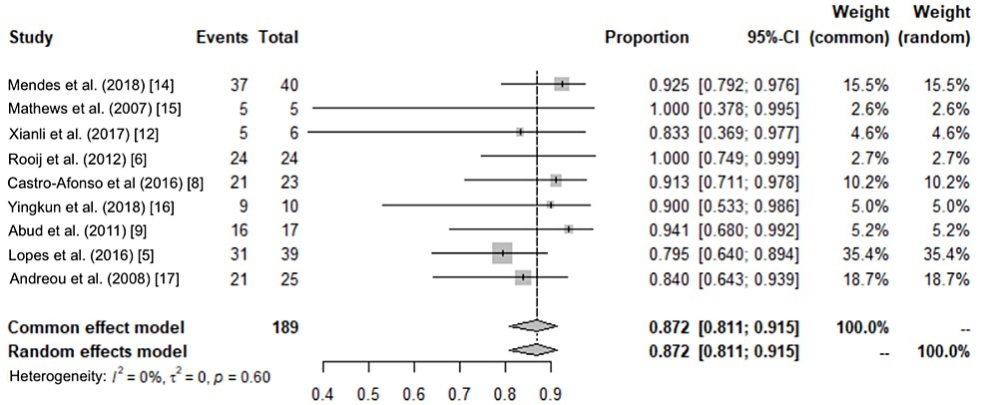
Forest plot after removing outliers

From the meta-analysis, we observed that I^2^ was 0% (0%, 64.8%) and tau-squared (estimate of between-study variance) was observed to be 0 (0, 1.1203). From Cochran’s Q test, we observe that there is no significant heterogeneity in the data (p-value = 0.6003). The following graph (Figure 4) shows the forest plot, which contains information on the heterogeneity test, proportions, and confidence intervals. 

**Figure 5:**
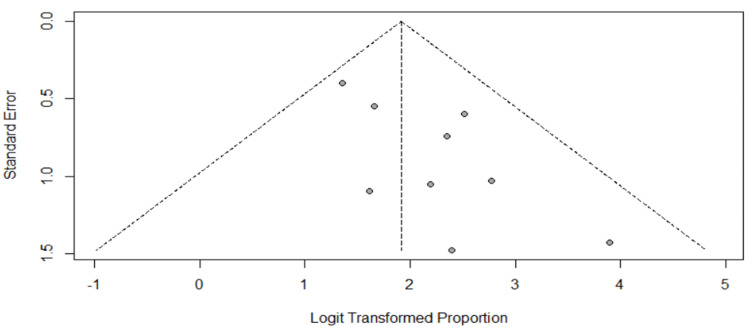
Funnel plot for publication bias

The overall proportion was 0.872 (0.811, 0.915). The following graph (Figure 5) gives the funnel plot.

From the funnel plot, we observe that all points are inside the funnel, indicating that there is a symmetric distribution of studies. Hence, we can say that there is no publication bias.

Note that Egger's test lacks the statistical power to detect bias when the number of studies is small (i.e., k<10). Hence, it was not reported.

The meta-analysis was done for complete cure rate proportions for the studies, which showed an overall 76% cure rate with a confidence interval of 95%. The publication bias was removed as mentioned earlier and regression analysis was then carried out.

The major complications observed in the studies under consideration were hemorrhage and neurodeficit, whose rates ranged from 0-0.20 and 0-0.16, respectively. The mean proportion for both major complications mentioned were 0.11 and 0.09, respectively. 

Long-term follow-up data were available in 11 studies; the rest of the studies had no mention of follow-up (Table 1). The range has been tabulated in Table 5.

**Table 5:** Range of long-term follow-up outcomes

Follow-up time period	Recurrence rate	Permanent disability rate
3 months	0-0.04	0.02-0.1
6 months	0-0.08	

The mortality rate was available only for two studies (Table 1) and ranged from 0.03 to 0.04.

Illustrative cases

Case 1

**Figure 6:**
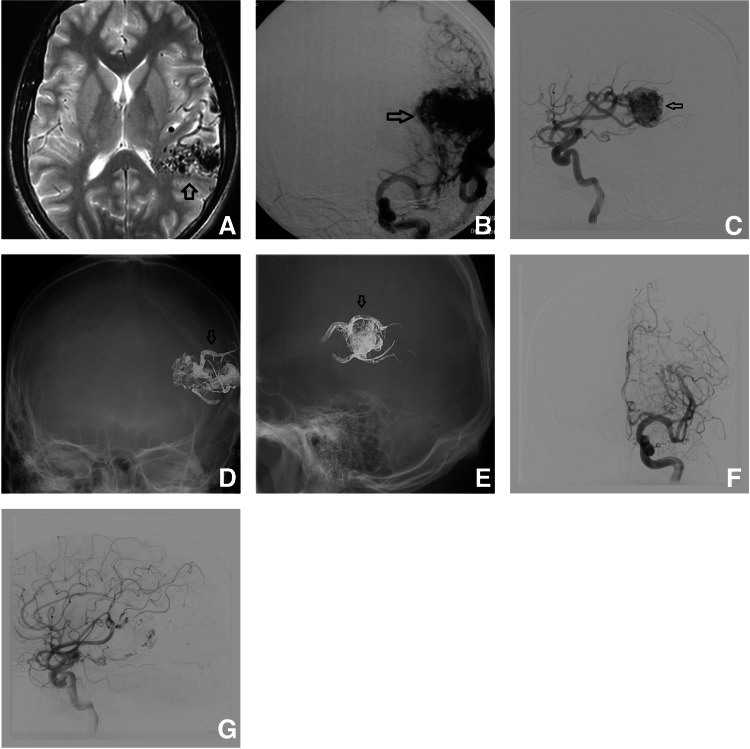
MRI shows an angular cortex compact nidus pointed at by an arrow (A), anteroposterior (B), and lateral (C) views of pre-embolization DSA shown by an arrow. (D) and (E) show anteroposterior and lateral views of Onyx (EVOH-based liquid embolizing agent) cast respectively marked by an arrow. Immediate post-embolization DSA shows in the anteroposterior (F) and lateral (G) views, showing complete obliteration of the AVM. Follow-up DSA after two years (not shown here) showed similar results. AVM - arteriovenous malformation; DSA - digital subtraction angiogram

A 38-year-old male presented with a headache. On radiological evaluation, a left cerebral AVM was diagnosed and therapeutic embolization was performed (Figure 6).

Case 2

**Figure 7:**
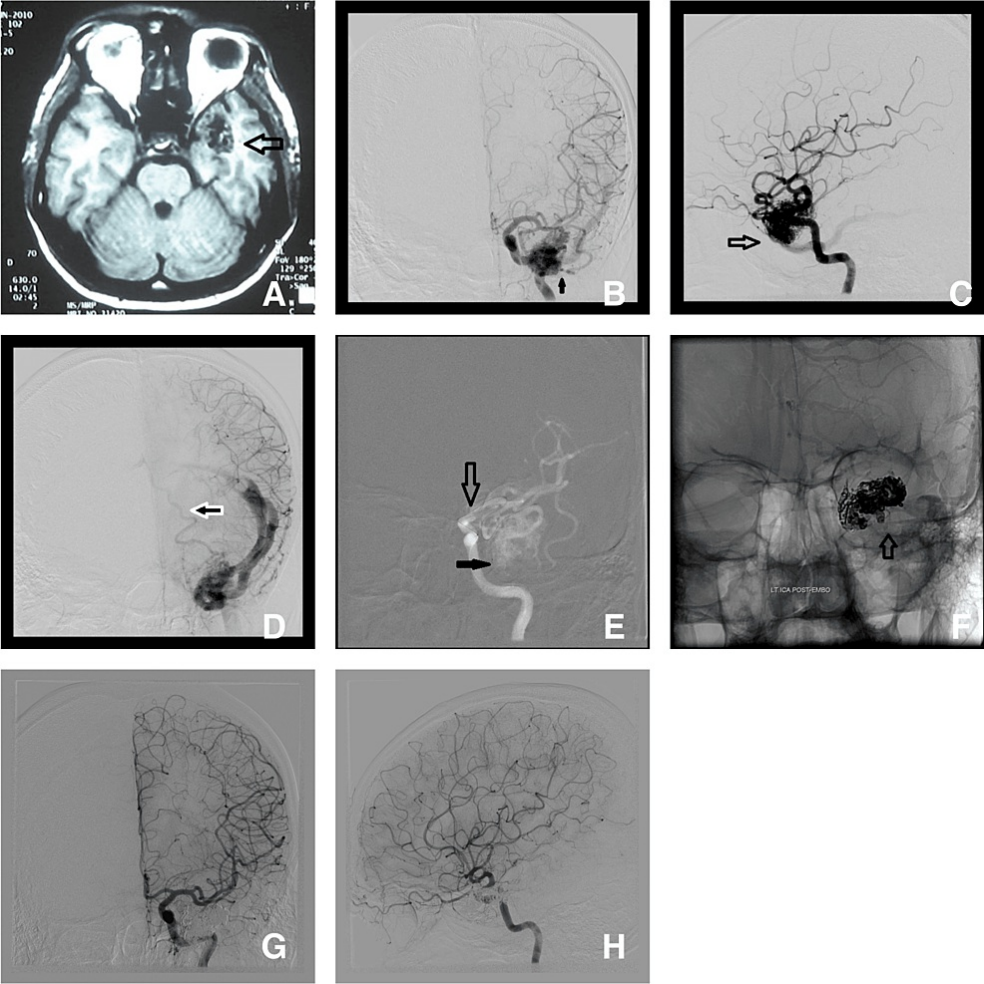
MRI axial plane (A) shows the left anterior temporal AVM. DSA images anteroposterior view (B) and lateral view (C) show the AVM nidus (empty black arrow). Image (D) anteroposterior view shows the deep Sylvian vein draining into the basal vein of Rosenthal (white-bordered black arrow). Image (E) shows the microcatheter taking multiple acute curves (empty black arrow) and the tip of the microcatheter just at the entrance of the nidus (solid black arrow). Image (F) anteroposterior view shows the Onyx cast. DSA anteroposterior view (G) and lateral view (H) show complete obliteration of the AVM in the immediate post-embolization stage. DSA after three years confirmed a complete cure (not shown here). AVM - arteriovenous malformation; DSA - digital subtraction angiogram

A 56-year-old female was diagnosed with left temporal AVM and embolization was done via the trans-arterial route (Figure 7).

Case 3

**Figure 8:**
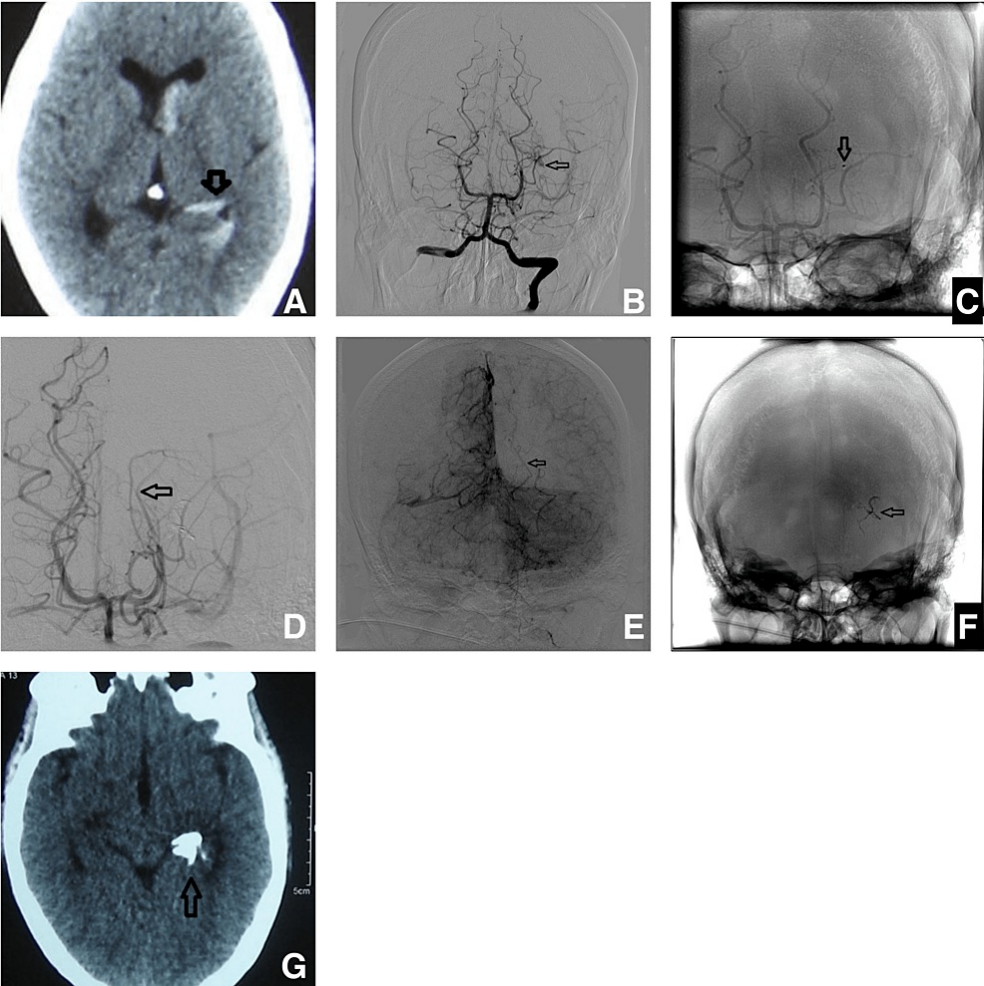
CT scan shows hemorrhage in the left choroid fissure with extension into the temporal and frontal horns of the lateral ventricle pointed by the arrow (A), DSA anteroposterior view (B) showing a small AVM nidus supplied by the posterolateral choroidal artery marked by the arrow. Fluoroscopic image (C) shows the catheter tip marked by the arrow. DSA image (D) shows complete exclusion of the AVM nidus. However, there is a non-visualization of the left distal posterior cerebral artery, which is reformed in the delayed phase (E); the arrow points to the left posterior cerebral artery, which appears to be in spasm. Fluoroscopic image (F) shows the Onyx cast marked by the arrow. Post-embolization CT shows the Onyx cast; note the streak of the cast in the choroid fissure marked by the arrow (G). A follow-up angiogram after one year showed no recurrence (not shown here). AVM - arteriovenous malformation; DSA - digital subtraction angiogram

A 32-year-old male presented with a headache and vomiting. Radiological evaluation revealed intra-ventricular and subarachnoid hemorrhage secondary to a small AVM (Figure 8). Trans-arterial embolization with Onyx was carried out.

Discussion

Neurosurgery has been established as the treatment of first choice for brain AVMs in the last century. Those inaccessible to surgery were subjected to radiosurgery. Since its inception, embolization has gone through turmoil, with regard to acceptance by the neurosurgery and neurology community for the management of AVMs. Its potential role as a standalone curative modality needs to be judiciously explored, as it can be offered for those AVMs inaccessible to surgery curing it in one or two sessions.

Evolution of the Philosophy of Embolization

Those AVMs that cannot be surgically cured were offered partial targeted embolization. However, it has been well established that partial embolization is futile and can be dangerous in the long term. Initially, the technique of embolization was not perfect with regards to the selection of approach, catheter technology, and embolizing agents. Additionally, the philosophy of treatment also evolved from targeted partial embolization to possible complete obliteration, wherein the prior technique is dangerous in the long term.

Angioarchitecture of AVMs and Grading System

Recent advances in imaging modality have helped us in understanding the morphology and hemodynamics of AVMs. 3T MRI with functional and dynamic angiographic capabilities, DSA with 3D/4D rotational angiography, and DSA-enabled cone beam CT mode have enabled us to visualize intra-nidal compartments, intranidal aneurysms, and venous outflow with detailed road mapping of the footsteps of primary veins and principle vein. These features are absolute prerequisites for better treatment planning, staging, and outcomes. The grading system is based on the Spetzler-Martin (SM) grading system, which estimates the risk of surgery, consisting of three elements - size, venous drainage, and location - is still valid. Ponce stratification and supplementary grading add further improvisations to SM grading and the Embocure score highlights the importance of angioarchitecture in treatment, planning, and outcomes [18-19].

In the coming paragraphs, a brief review of general guidelines, embolization techniques, and various improvised embolization methods mentioned in the articles under review is reported.

Embolization Technique and General Guidelines

Unilateral 6F transfemoral access is preferably gained under ultrasound (USG) guidance. Systemic heparinization was given at a dosage of 3000 IU - loading and 1000 IU - maintenance per hour thereafter [20]. 6F Guiding catheter (Envoy, Codman Neuro, Raynham, MA) was navigated and parked in the target artery. Supple flow guided micro-catheter (Ultraflow or Apollo ev3, Irvine, California, Medtronics or Sonic, Balt, Montmorency, France) was navigated into the feeding artery (in case of two feeders, wherever the micro-catheter goes first) with its tip placed as close to the nidus as possible, preferably at the entrance of the nidus. A super-selective angiogram was taken to further characterize the angioarchitecture. The microcatheter was flushed with normal saline. It was filled with dimethyl sulfoxide (DMSO) up to the dead space, typically 0.3 ml over 45 seconds. The EVOH (Onyx-18, Medtronic, Cork, Ireland) and Squid 12 or 18 (Balt, Montmorency, France)] embolizing agent was slowly injected over the blank roadmap at the speed of 0.1 ml over 1-2 minutes. The total volume of liquid embolic agent injected was 2-3 vials [21]. The duration of the injection ranged from 40 minutes to 60 minutes. The target was to completely obliterate the nidus. The EVOH should spread from the feeder artery toward the vein and centrifugally. Care was taken to reach the footsteps of veins at the end and finally occluding the venous drainage along with the complete exclusion of the nidus from the circulation. Heparin was then reversed. Then with a gentle pull, the catheter was snapped out. Detachable tip micro-catheters were always preferred whenever available. We should take utmost precaution that EVOH should spread from the catheter tip toward the footsteps of veins and centrifugally. If EVOH is reaching veins before completely obliterating the nidus, we should either stop and plan for a second session at a later date or try injecting from the other feeder. But premature closure of the draining vein should always be avoided. If it happens then surgical excision should be considered as soon as possible to safeguard from delayed hemorrhage.

Endovascular embolization of brain AVMs has evolved significantly in the last decade with the availability of ethyl vinyl alcohol copolymer (EVOH) [22]. The advantage of EVOH is that it is nonadhesive in nature as compared to n-butyl cyanoacrylate glue (NBCA). A prolonged injection is possible without the microcatheter getting stuck, which facilitates complete obliteration of the nidus in a single session. Detachable micro-catheter tips add further safety over non-detachable tips if the catheter gets stuck in the EVOH cast; it can still be pulled out with fewer chances of vessel injury.

Schedule of the Embolization

Ruptured AVMs need to be treated as soon as possible, as re-bleeding cannot be predicted. Most of the time, small AVMs can be obliterated completely in a single session. If for some reason, a single session was not possible, compartments to be embolized in the first session should be planned beforehand, sparing the draining vein.

Special Techniques of Embolization

1. Double catheter technique (DACT): The DACT technique involves putting two catheters in two different feeders and alternatively or simultaneously performing embolization of the nidus. It has the potential to reduce the embolization time and radiation dose, and increase the chances of complete obliteration of AVMs [23].

2. Pressure cooker: The pressure cooker technique (PCT) was designed to create an anti-reflux plug by trapping the detachable part of an Onyx-compatible microcatheter with coils and glue in order to obtain wedge-flow conditions, thereby enabling more comprehensive, forceful, and controlled EVOH embolization. The PCT might enlarge the range of AVMs amenable to endovascular cure [24].

3. Transvenous approach: Transvenous embolization is considered a salvage therapy in contemporary AVM management. Proposed indications for this approach include a small (diameter < 3 cm) and compact AVM nidus, deep AVM location, hemorrhagic presentation, single draining vein, lack of an accessible arterial pedicle, exclusive arterial supply by perforators, and en-passage feeding arteries. Available studies of transvenous AVM embolization in the literature have reported high complete obliteration rates, with reasonably low complication rates. However, evaluating the efficacy and safety of this approach is challenging due to the limited number of published cases.

Complications of Embolization

Complications can be ischemic or hemorrhagic. There is ischemic damage to normal brain parenchyma in the immediate vicinity of the nidus or compression by the onyx cast. Unintentional, excessive reflux of EVOH resulting in occlusion of normal branches or toxicity of DMSO due to high volume should be avoided. Hemorrhage can be due to perforation of an artery from protruding microwire, avulsion of the cortical branch during withdrawal of the catheter after embolization, or delayed hemorrhage due to venous occlusion. Avoiding protrusion of the microwire beyond the catheter tip during navigation as much as possible and using a detachable tip catheter [24] might help in reducing hemorrhagic complications. Our review suggests major complication rates range from 0-20%, which includes a permanent disability rate of 2-10% and recurrence of 4-8%. The mortality rate ranged from 3-4%. The complication rates for microsurgical excision ranged from 20-60% in the available literature, whereas complication rates for radiosurgery were not mentioned in the available literature.

Post-embolization Care

Post-procedure patients should be extubated in the cath-lab while taking care not to cause hypertension during extubation. Patients should be kept in an ICU under strict observation with target BP maintained below 110-120 systolic and below 70-80 diastolic. Lower ranges of blood pressure were preferred [17].

Outcome Measures and Follow-Ups

Outcomes are to be evaluated by DSA immediately after embolization and any time between one to three years thereafter. Clinical outcomes are to be evaluated by a modified Rankin scale (MRS) before discharge and on subsequent follow-up at one month, six months, and every year thereafter. Anti-epileptic drugs should be started prior to the procedure and continue to be rewarded [23-24]. However, the long-term use of anti-epileptics was not required in most of the cases.

What to Choose From: Embolization, Neurosurgery, Radiosurgery, or Medical Management [20]?

Choice of treatment should always involve a multidisciplinary discussion. Neurosurgery and embolization have curative potential but have increased complications as compared to radiosurgery. All the options should be discussed with the patient and the most suitable modality has to be chosen for a given case, after discussing all the parameters.

## Conclusions

Embolization of small (Spetzler-Ponce grade A) brain AVMs with an intention to cure by a non-adhesive liquid embolic agent can deliver a complete obliteration/cure rate of 76% with a CI of 95%. Major complication rates associated with embolization ranged from 0 to 20 %, with the permanent disability rate being 2-10%, recurrence of 4-8%, and mortality of 3-4%. However, prospective registries and randomized control trials involving embolization versus neurosurgery and/or stereotactic radiosurgery (SRS) may validate the role of embolization in small brain AVMs as a curative treatment.
